# Circulating sphingolipids and subclinical brain pathology: the cardiovascular health study

**DOI:** 10.3389/fneur.2024.1385623

**Published:** 2024-05-03

**Authors:** Kristine F. Moseholm, Jens W. Horn, Annette L. Fitzpatrick, Luc Djoussé, W. T. Longstreth, Oscar L. Lopez, Andrew N. Hoofnagle, Majken K. Jensen, Rozenn N. Lemaitre, Kenneth J. Mukamal

**Affiliations:** ^1^Department of Public Health, Section of Epidemiology, University of Copenhagen, Copenhagen, Denmark; ^2^Department of Internal Medicine, Levanger Hospital, Health Trust Nord-Trøndelag, Levanger, Norway; ^3^Departments of Family Medicine and Epidemiology, School of Public Health, University of Washington, Seattle, WA, United States; ^4^Division of Aging, Department of Medicine, Brigham and Women’s Hospital and Harvard Medical School, Boston, MA, United States; ^5^Department of Nutrition, Harvard T.H. Chan School of Public Health, Boston, MA, United States; ^6^Department of Neurology, School of Medicine, University of Washington, Seattle, WA, United States; ^7^Department of Neurology, School of Medicine, University of Pittsburgh, Pittsburgh, PA, United States; ^8^Department of Laboratory Medicine and Pathology, School of Medicine, University of Washington, Seattle, WA, United States; ^9^Cardiovascular Health Research Unit, Department of Medicine, University of Washington, Seattle, WA, United States; ^10^Department of Medicine, Beth Israel Deaconess Medical Center, Boston, MA, United States

**Keywords:** white matter hyperintensity, ventricular size, hippocampal atrophy, brain infarcts, neurofilament light chain, glial fibrillary acidic protein, ceramide, sphingomyelin

## Abstract

**Background:**

Sphingolipids are implicated in neurodegeneration and neuroinflammation. We assessed the potential role of circulating ceramides and sphingomyelins in subclinical brain pathology by investigating their association with brain magnetic resonance imaging (MRI) measures and circulating biomarkers of brain injury, neurofilament light chain (NfL) and glial fibrillary acidic protein (GFAP) in the Cardiovascular Health Study (CHS), a large and intensively phenotyped cohort of older adults.

**Methods:**

Brain MRI was offered twice to CHS participants with a mean of 5 years between scans, and results were available from both time points in 2,116 participants (mean age 76 years; 40% male; and 25% *APOE* ε4 allele carriers). We measured 8 ceramide and sphingomyelin species in plasma samples and examined the associations with several MRI, including worsening grades of white matter hyperintensities and ventricular size, number of brain infarcts, and measures of brain atrophy in a subset with quantitative measures. We also investigated the sphingolipid associations with serum NfL and GFAP.

**Results:**

In the fully adjusted model, higher plasma levels of ceramides and sphingomyelins with a long (16-carbon) saturated fatty acid were associated with higher blood levels of NfL [β = 0.05, false-discovery rate corrected *P* (*P_FDR_*) = 0.004 and β = 0.06, *P_FDR_* = < 0.001, respectively]. In contrast, sphingomyelins with very long (20- and 22-carbon) saturated fatty acids tended to have an inverse association with levels of circulating NfL. In secondary analyses, we found an interaction between ceramide d18:1/20:0 and sex (*P* for interaction = <0.001), such that ceramide d18:1/20:0 associated with higher odds for infarcts in women [OR = 1.26 (95%CI: 1.07, 1.49), *P_FDR_* = 0.03]. We did not observe any associations with GFAP blood levels, white matter grade, ventricular grade, mean bilateral hippocampal volume, or total brain volume.

**Conclusion:**

Overall, our comprehensive investigation supports the evidence that ceramides and sphingomyelins are associated with increased aging brain pathology and that the direction of association depends on the fatty acid attached to the sphingosine backbone.

## Introduction

Dementia is a major global public health emergency, and identifying its pathophysiological risk factors remains a key scientific priority (1–3). Subclinical vascular brain injury such as covert infarction and white matter changes contribute to cognitive decline and dementia (4–7). Additionally, cerebral small vessel disease (CSVD) has emerged as a major cause of stroke and the leading contributor to vascular dementia (8).

Sphingolipids are a group of lipids characterized by a sphingosine backbone to which a fatty acid is acylated. They are especially concentrated in myelin sheath in the brain and relay important signals for intra- and intercellular events (9–11). Circulating levels of sphingolipids have been associated with both cerebrovascular and neurodegenerative diseases such as stroke and dementia (12–14). One family of sphingolipids, the ceramides (Cer) has been shown to accumulate in tissues such as the liver and brain during aging (15–17), and emerging evidence indicates that Cer are the most dysregulated lipids in cerebrovascular diseases, including stroke and CSVD (18–21). Blood levels of Cer and sphingomyelins (SM), another class of sphingolipids that can be metabolized to Cer, have both been associated with hippocampal atrophy, white matter hyperintensities, and white matter microstructural changes (22–24).

Cer and SM with different fatty acid chain lengths may exhibit distinct biological activities (25, 26). Indeed, we have shown that higher levels of circulating Cer and SM with a saturated fatty acid chain length of 16-carbon (Cer-16 and SM-16) are directly associated with cardiovascular disease and mortality, while circulating Cer and SM with 22- and 24-carbon saturated fatty acid chain length (Cer-22, Cer-24, SM-22, SM-24) are inversely associated (27–29).

Here, we address the role of Cer and SM in subclinical brain pathology by investigating their association with a broad range of measures in the Cardiovascular Health Study (CHS), a large and extensively phenotyped cohort of older adults. Based on previous observations, we hypothesize that Cer and SM with different saturated fatty acids will differ in associations, thus this study focus on 4 Cer species with the fatty acids palmitic acid, arachidic acid, behenic acid and lignoceric acid (Cer-16, Cer-20, Cer-22, Cer-24, respectively) and 4 analogous SM species (SM-16, SM-20, SM-22, SM-24). We examined several magnetic resonance imaging (MRI) measurements such as white matter hyperintensities, ventricular size, and brain atrophy, and two circulating biomarkers of brain injury, neurofilament light chain (NfL) and glial fibrillary acidic protein (GFAP), to gain a comprehensive view of the associations of 8 sphingolipid species with these measures.

## Methods

### Study population

**Figure 1:**
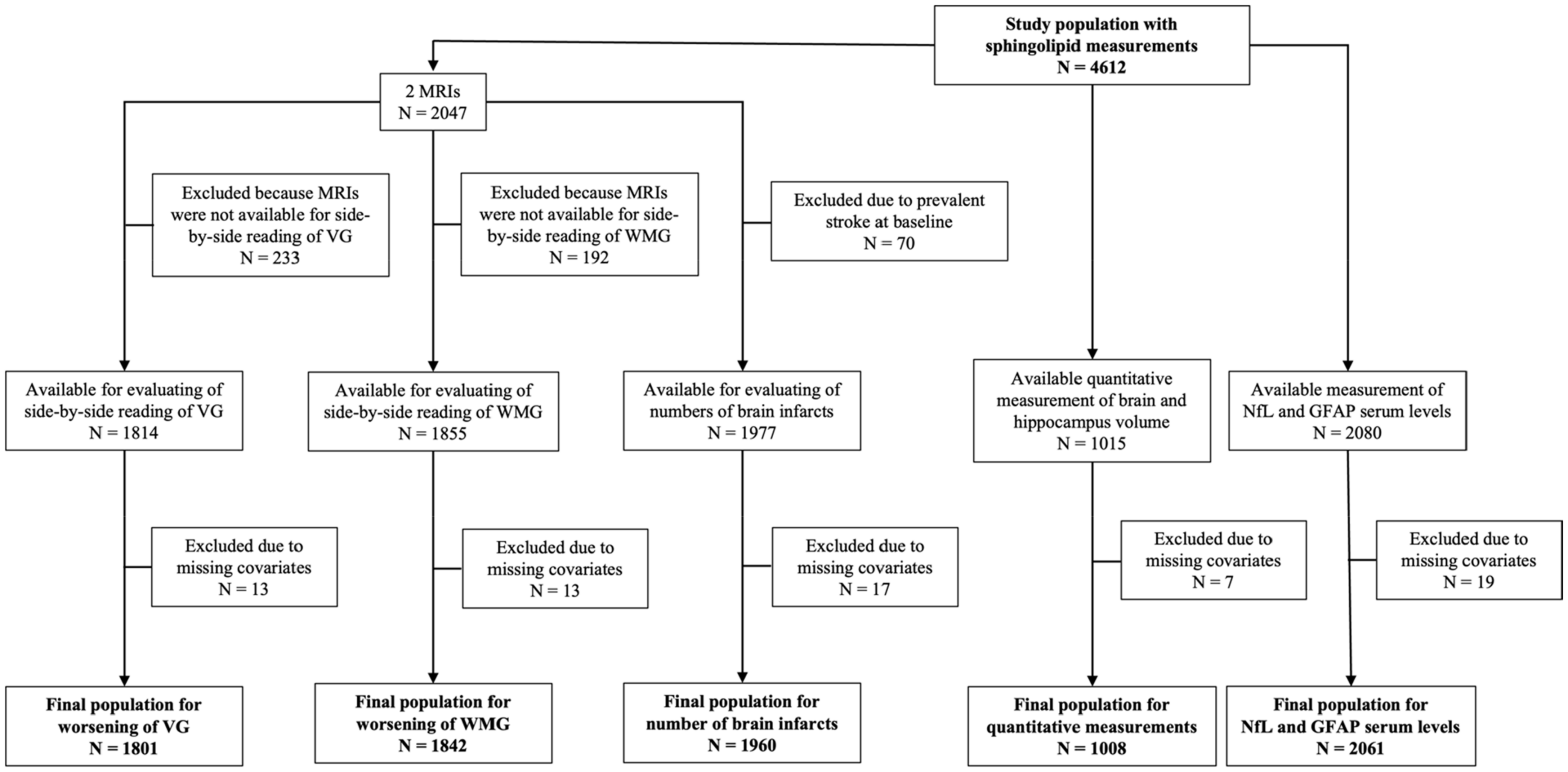
Flowchart of study populations. GFAP, glial fibrillary acidic protein; MRI, magnetic resonance imaging; NfL, neurofilament light chain; VG, ventricular grade; WMG, white matter grade.

The CHS began in 1989–1990 and recruited 5,201 women and men aged 65 years in 4 different US communities: Forsyth County, NC; Sacramento County, CA; Washington County, MD; Allegheny CA. In 1992–1993, an additional 687 predominantly African American participants were recruited in 3 of the 4 original communities. Potential participants were randomly identified from Medicare eligibility lists. The study design and sampling methods have been previously described in detail (30). During the first decade of the study, participants underwent annual clinic visits, followed by semiannual phone contacts thereafter. Each clinic visit involved comprehensive physical examinations and the collection of demographics, anthropometry, blood pressure, psychosocial interviews, depression assessments, medical history, health behaviors, physical function evaluations, hematology, laboratory examinations, and medication records. For the present analyses, sphingolipids were measured in plasma samples from 586 participants from 1992 to 1993 visit and 4,026 participants from the 1994–1995 visit (total *N* = 4,612). The clinic visit from which sphingolipids were measured (1992–1993 or 1994–1995) formed the study baseline in the analyses. Figure 1 shows the flow and number of participants included in each of our analyses.

### Measurements of sphingolipids

Measurement of sphingolipid species were performed using EDTA-plasma samples that had been stored at −70°C. Plasma lipids were extracted, and sphingolipids quantified by liquid chromatography–tandem mass spectrometry. Previous work suggests that sphingolipid species are robust to storage at −70°C (31). A comprehensive description of the methods and quality control for sphingolipid measurement, including details on lipid extraction, sample plating, instrumentation, internal standards, and normalization, can be found in previous publications (27, 32). In the current study, we restricted analyses to 8 primary sphingolipids. This included 4 Cer species with the fatty acids palmitic acid, arachidic acid, behenic acid and lignoceric acid (Cer-16, Cer-20, Cer-22, Cer-24, respectively) and 4 analogous SM species (SM-16, SM-20, SM-22, SM-24).

### Brain imaging

Brain MRI was offered twice to CHS participants in 1992–1994 and 1997–1999, as described earlier (33, 34). Results were available from both time points in 2,116 participants with a mean of 5 years between scans. Non-contrast MRI imaging included sagittal T1-weighted, spin-density, and T2-weighted images with 5-mm thickness and no interslice gaps (35). At a single reading center, neuroradiologists rated the MRI scans blinded to study information (33).

#### White matter hyperintensities

White matter hyperintensities (WMH) were identified on either axial T2-weighted or spin-density images scans and graded on a similar 10-point semiquantitative white matter grade (WMG), ranging from 0 (least) to 9 (most abnormal). WMG had an inter-reader interclass kappa correlation coefficient of 0.76 and intra-reader kappa coefficient of 0.89 (36, 37). To assess white matter grades over time, we used MRIs eligible for side-by-side re-reading. Because of technical problems, 197 of the original 2,116 pairs of scans could not be re-read, leaving 1,919 (91%) pairs (38). To document worsening WMG of 1 or more grades (yes vs. no), the neuroradiologists read all scans from the same participant side-by-side without knowledge of which scan was performed first (38).

#### Ventricular grade

Ventricular sizes were estimated from the T1-weighted axial images. The size of the lateral ventricles was estimated by the radiologist based on a referenced standard to create a ventricular grade (VG) ranging from 0 (least) to 9 (most abnormal) (39). Intra-reader agreement within 1 grade was 94% with a kappa of 0.89 for the VG (39). Worsening of VG by one or more grades (yes vs. no) was determined by neuroradiologists in side-by-side reading without knowledge of which scan was performed first.

#### Total brain volume and mean bilateral hippocampi volume

A subgroup of the follow-up MRIs was performed using a 1.5-tesla scanner and included 3D T1-weighted spoiled gradient-recall sequences, as described previously (40). These scans were processed with FreeSurfer software, which converted high-resolution T1-weighted images into 1x1x1 mm3 voxels and generated a surface-based reconstruction of the brain to estimate the quantitative volume of the hippocampus and total intracranial space, which were then converted to normalized brain volume (1-NBV) units. Volumetric measurements from the right and left hippocampus were averaged to compute the mean bilateral hippocampal volume.

#### Brain infarcts

Brain infarcts were defined as abnormal signal intensities 3 mm in size or larger, identified by neuroradiologists on MRI scans. Number of brain infarcts was defined as one or more new infarcts on the follow-up MRI.

### Quantification of blood NfL and GFAP

Frozen fasting serum samples collected from participants at the 1996–1997 clinic visit were used to measure NfL and GFAP. The CHS Central Laboratory at the University of Vermont measured NfL and GFAP using the 4th generation single-molecule array Simoa™ Human Neurology 4-Plex A assay (N4PA, Quanterix™). As the measurements were funded as part of a metabolic ancillary study to CHS, they were limited to participants who underwent an oral glucose tolerance test that excluded participants with treated diabetes. The inter-assay coefficients of variation were 9.3% for NfL and 8.2% for GFAP.

### Covariates

Information on age (years), race/ethnicity (Caucasian, African American), education (years), physical activity (kcal/week), alcohol intake (drinks/week), smoking status (never, former, current), medication-use, and CESD Depression score was based on self-report. Weight, height, and blood pressure were measured using standardized protocols. Body mass index (BMI) was calculated as weight in kilograms divided by height in meters squared (kg/m^2^). Hypertension was defined by systolic blood pressure ≥ 140 mm Hg or diastolic blood pressure ≥ 90 mm Hg or use of antihypertensive medication. Plasma lipids and glucose were measured on fasting blood samples using standard protocols (30). Prevalent diabetes mellitus was defined by a fasting glucose concentration of ≥7 mmol/L (126 mg/dL) or use of insulin or oral hypoglycemic agents. History of stroke and coronary heart disease was obtained from medical records, participant and/or proxy reports and adjudicated by committee (41, 42).

### Statistical analysis

Baseline characteristics were presented for continuous variables as mean (SD) and for categorical variables as numbers (percentages). Sphingolipid species concentrations were log-transformed to reduce skewness. We used non-parametric testing to estimate correlations between sphingolipid species.

Logistic regression was used to assess associations with the dichotomous outcomes of VG and WMG worsening on follow-up MRI, using each sphingolipid specie concentration as a continuous variable [per one standard deviation (SD) in log sphingolipid species concentration (μM)]. We used ordinal logistic regression to model the associations with number of infarcts, where the number of infarcts was categorized as 0, 1 to 2, and ≥3 based on evaluation of the proportional odds assumption using a score test. We excluded all participants with prevalent stroke (*N* = 70) at baseline for this analysis. These analyses were initially adjusted for age (years), sex, race/ethnicity (Caucasian, African American), education (years), field center (4 sites), and baseline year (1992–1993 or 1994–1995) (Model 1). Next, we additionally included BMI (kg/m^2^), physical activity (kcal/week), alcohol intake (drinks/week), smoking status (never, former, current), depression score (continuous), diabetes (yes/no), coronary heart disease (yes/no), hypertension (yes/no), lipid-lowering medication use (yes/no), HDL-cholesterol (mg/dL), and LDL-cholesterol (mg/dL) (Model 2). All covariates were assessed at the respective baseline visit. Due to mutual correlation and contrasting biological properties, a third model was included in which Cer/SM with an acylated 16-carbon fatty acid were adjusted for Cer/SM with 22-carbon fatty acid, and Cer/SM with 20-, 22-, 24-carbon fatty acids were each adjusted for Cer/SM with a 16-carbon fatty acid (Model 3). This approach has been reported elsewhere (27–29, 43).

The associations with total brain volume and mean bilateral hippocampi volume were assessed using linear regression, adjusted for the same covariates as above and estimated total intracranial volume. The associations with circulating NfL and GFAP were also analyzed using linear regression models. Due to skewness, we winsorized 10 GFAP outliers to the value of 1,000 pg./mL and 9 NfL outliers to the value of 250 pg./mL and transformed their values to the natural logarithm (log_n_Nfl or log_n_GFAP).

In secondary analyses, we tested for interactions with sex and race/ethnicity of the associations of sphingolipids with brain pathology by inclusion of multiplicative interaction terms in the respective models. Lastly, we performed stratified analyses based on *APOE* genotype, as *APOE*ε*4* carriers have increased risk of development of neurodegenerative diseases such as dementia. Analyses were limited to those with available DNA who consented to genetic studies.

To correct the analyses for multiple comparisons, we applied a false discovery rate (FDR) *p*-value correction in which *p* = 0.05 was considered significant. All statistical analyses were conducted using R version 4.1.3 (44).

## Results

Figure 1 shows the number of CHS participants in each analysis, based upon outcome and exclusions ranging from *N* = 1,008 for quantitative analyses of brain volumes to *N* = 2,061 for circulating NfL and GFAP. No major differences in population characteristics between the five groups included in the different analyses were observed. Mean age was 76 years, and 57.9% to 60.4% of participants were female and 12.6% to 15.7% were African American, depending on the analyses (Supplementary Table S1). The measured sphingolipid species showed moderate inter-correlations, with stronger correlations observed between sphingolipids with very long chain fatty acid (20-, 22-, and 24-carbon) and coefficients of variation estimated over the whole study period ranged from 5.9 to 18.6% (Supplementary Table S2).

### No associations with volumetric measurements, WMG, or VG worsening over 5 years

We observed no clear associations for any of the sphingolipid species with worsening in WMG, decrease in total brain volume, or mean bilateral hippocampal volume. In the fully adjusted model, elevated plasma levels of Cer-16 and SM-16 were associated with higher odds of worsening in VG, but this finding was not statistically significant upon *p*-value correction. In contrast, Cer-22 was associated with lower odds of worsening in VG, which also became non-significant upon *p*-value correction (data not shown).

### Cer-20 associates with higher number of brain infarcts in women

**Figure 2:**
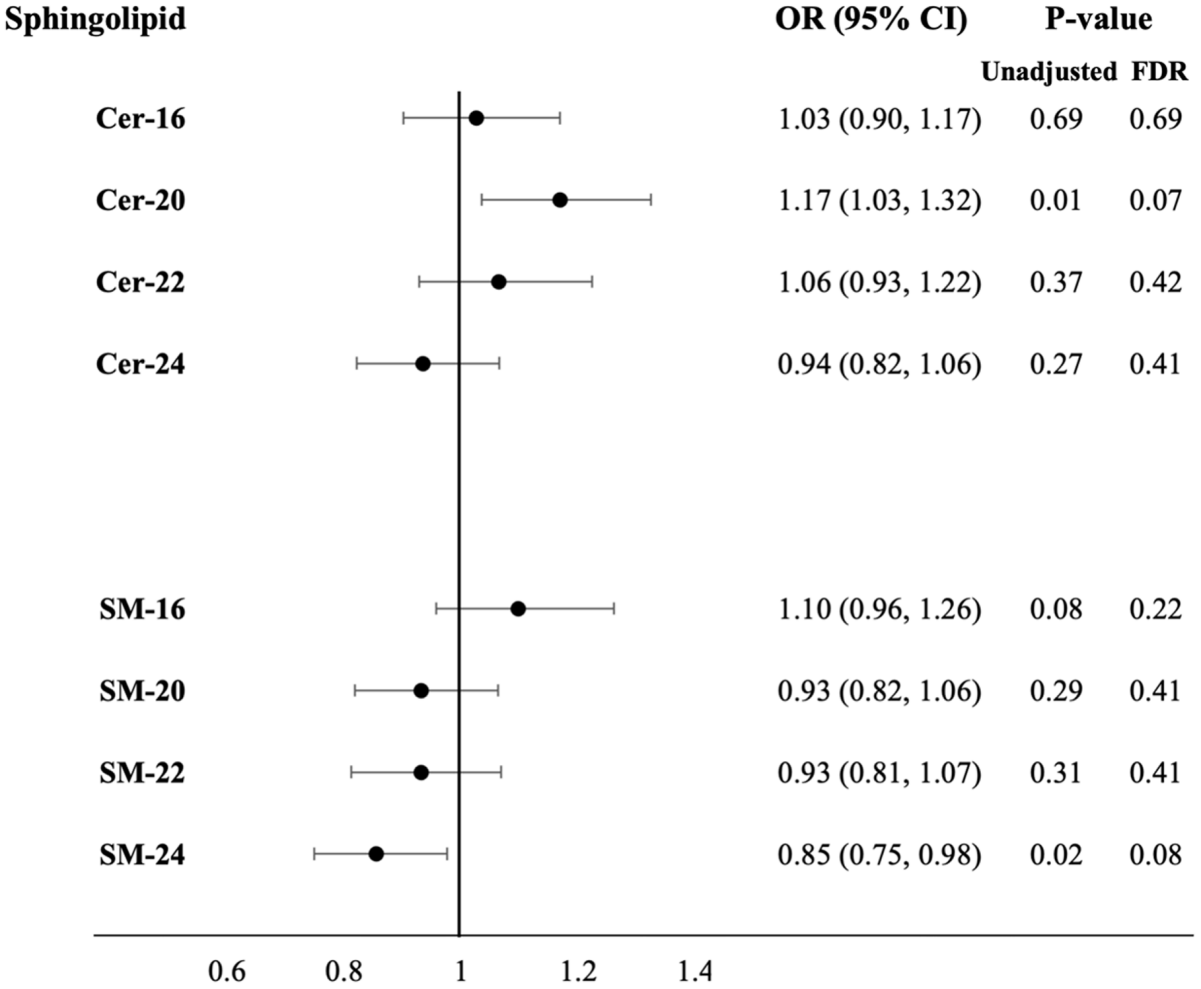
Adjusted odds ratios for increase in number of brain infarcts per SD higher plasma log-sphingolipid concentrations, *N* = 1,960. Cer, ceramide; CI, confidence interval; FDR, false discovery rate; OR, odds ratio; SD, standard deviation; SM, sphingomyelins; 16, 20, 22, and 24 stand for the number of carbons of the saturated fatty acid acylated to the sphingolipid backbone. Odds ratios adjusted for age, sex, race/ethnicity, education, field center, year of blood sample measurement, BMI, physical activity, alcohol intake, smoking status, depression score, prevalent diabetes, prevalent coronary heart disease, prevalent hypertension, lipid-lowering medication use, HDL-cholesterol, LDL-cholesterol and mutual adjustment of sphingolipids with 16-carbon fatty acid chains with corresponding sphingolipids with 22-carbon fatty acid chains and sphingolipid with 20-, 22-, 24-carbon fatty acid chains with sphingolipids with 16-carbon fatty acid chains (Model 3).

Higher plasma levels of Cer-20 associated with increased odds for more brain infarcts in Model 1 [OR = 1.18 (1.07, 1.31), *P_FDR_* = 0.008] and 2 [OR = 1.16 (1.04, 1.29), *P_FDR_* = 0.03], which attenuated upon p-value correction in Model 3 (OR = 1.17 (1.03, 1.32), *P_FDR_* = 0.07; Figure 2; Supplementary Table S3 for estimates of Model 1 and Model 2). In sex-stratified analysis, Cer-20 was associated with 26% higher odds [OR = 1.26 (1.07, 1.49), *P_FDR_* = 0.03, *P* for interaction = <0.001] in women and not in men (Table 1).

**Table 1:** Adjusted odds ratios for increase in number of brain infarcts per SD higher plasma log-sphingolipid concentrations in sex-stratified analysis.

Sphingolipid	OR for increase in number of brain infarcts (95% CI)					
	Women (*N* = 1,175)			Men (*N* = 785)		
	Model 3	*P*-value		Model 3	*P*-value	
		*Unadjusted*	*FDR*		*Unadjusted*	*FDR*
Cer-16	0.97 (0.82, 1.15)	0.74	0.82	1.11 (0.90, 1.36)	0.34	0.75
Cer-20	1.26 (1.07, 1.49)	0.004	0.03	1.06 (0.88, 1.28)	0.50	0.75
Cer-22	1.14 (0.96, 1.37)	0.14	0.22	0.96 (0.77, 1.20)	0.75	0.75
Cer-24	0.98 (0.83, 1.16)	0.82	0.82	0.87 (0.71, 1.07)	0.14	0.75
SM-16	1.15 (0.96, 1.37)	0.05	0.10	1.03 (0.82, 1.29)	0.71	0.75
SM-20	0.83 (0.69, 0.99)	0.03	0.09	1.09 (0.89, 1.34)	0.40	0.75
SM-22	0.92 (0.77, 1.10)	0.38	0.50	0.94 (0.75, 1.17)	0.57	0.75
SM-24	0.82 (0.70, 0.98)	0.03	0.09	0.90 (0.73, 1.12)	0.34	0.75

Cer, ceramide; CI, confidence interval; FDR, false discovery rate; OR, odds ratio; SM, sphingomyelins; 16, 20, 22, and 24 stand for the number of carbons of the saturated fatty acid acylated to the sphingolipid backbone. Model 3 adjusted for age, sex, race/ethnicity, education, field center, year of blood sample measurement, BMI, physical activity, alcohol intake, smoking status, depression score, prevalent diabetes, prevalent coronary heart disease, prevalent hypertension, lipid-lowering medication use, HDL-cholesterol, and LDL-cholesterol, mutual adjustment of sphingolipids with 16-carbon fatty acid chains with sphingolipids with 22-carbon fatty acid chains and sphingolipids with 20-, 22-, 24-carbon fatty acid chains with sphingolipids with 16-carbon fatty acid chains. Cer-20, *P* for sex interaction = <0.001.

### Cer-16 and SM-16 associates with higher circulating NfL levels

**Figure 3:**
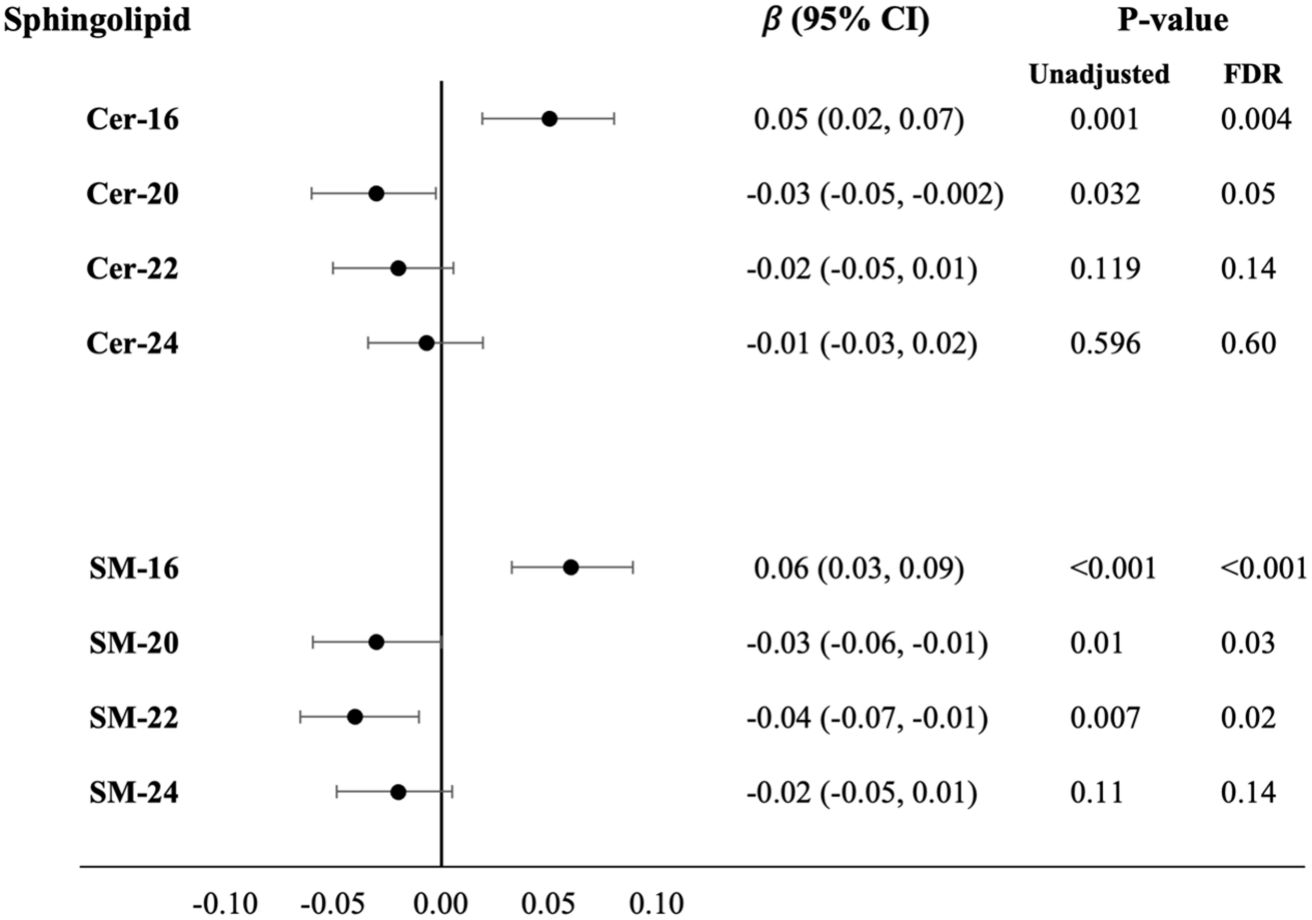
Cross-sectional associations with difference in serum NfL levels per SD higher plasma log-sphingolipid concentrations, *N* = 2,061. Cer, ceramide; CI, confidence interval; FDR, false discovery rate; NfL, neurofilament light chain; SD, standard deviation; SM, sphingomyelins; 16, 20, 22, and 24 stand for the number of carbons of the saturated fatty acid acylated to the sphingolipid backbone. Linear regression beta coefficients adjusted for age, sex, race/ethnicity, education, field center, year of blood sample measurement, BMI, physical activity, alcohol intake, smoking status, depression score, prevalent diabetes, prevalent coronary heart disease, prevalent hypertension, lipid-lowering medication use, HDL-cholesterol, LDL-cholesterol and mutual adjustment of sphingolipids with 16-carbon fatty acid chains with corresponding sphingolipids with 22-carbon fatty acid chains and sphingolipid with 20-, 22-, 24-carbon fatty acid chains with sphingolipids with 16-carbon fatty acid chains (Model 3).

Higher plasma levels of Cer and SM with long fatty acids were associated with higher levels of circulating NfL: Cer-16 (β = 0.05, *P_FDR_* = 0.004) and SM-16 (β = 0.06, *P_FDR_* = <0.001). In contrast, higher levels of several Cer and SM with very long chain fatty acids were associated with lower levels of circulating NfL; Cer-20 (β = −0.03, *P_FDR_* = 0.05), SM-20 (β = −0.03, *P_FDR_* = 0.03), and SM-22 (β = −0.04, *P_FDR_* = 0.02; Figure 3; Supplementary Table S4 for estimates of Model 1 and Model 2). In *APOE* genotype stratified analysis, higher levels of SM-16 in *APOE ε4* carriers associated with higher levels of circulating NfL (β = 0.07, *P_FDR_* = 0.03), whereas the association was not statistically significant in *APOE ε4* non-carriers (β = 0.03, *P_FDR_* = 0.22; Supplementary Table S5). For associations with levels of circulation GFAP, no associations were observed after covariate adjustments and *p*-value correction.

## Discussion

Overall, our comprehensive investigation supports the evidence that Cer and SM species with a bound (acylated) 16-carbon long fatty acid are associated with increased signs of degenerative changes on MRI. In this prospective study in older adults, higher plasma levels of Cer-16 and SM-16 were associated with higher blood levels of NfL, a biomarker of brain injury, and tended to associate with worsening of VG over 5 years, however, non-significant upon adjustment for multiple comparisons. In *APOE ε4* carriers, higher levels of SM-16 associated with higher levels of circulating NfL, and no associations were observed in *APOE ε4* non-carriers. Additionally, Cer and SM with a very long (20- and 22-carbon) chain fatty acids show an inverse association with levels of circulating NfL. However, Cer-20 also associated with higher odds for infarcts in women in secondary analyses. We did not observe any associations with levels of GFAP, WMG or mean bilateral hippocampal volume.

Previous epidemiological studies have found that higher plasma levels of Cer-16 associate with greater WMH volumes (23) and higher plasma levels of Cer-22 and Cer-24 associate with greater right hippocampal volume loss 1 year later in 17 patients with amnesiac mild cognitive impairment (22). We did not confirm either of these associations, although we did find that Cer-16 appears to be associated with ongoing brain injury and global atrophy. Part of the difference in these results may relate to the prospective nature of our white matter findings, which specifically examined progression late in life. Another possibility might be that Cer-16 confounded the associations of Cer-22 and Cer-24.

To the best of our knowledge, our results are novel, as no previous study has reported on the associations of sphingolipids and VG or blood levels of NfL. Neuronal cell death is a hallmark of brain atrophy, including ventricular enlargement. Ceramides have been implicated in promoting apoptosis and neuronal loss through various mechanisms, including mitochondrial dysfunction, activation of pro-apoptotic pathways, and disruption of neuronal survival signaling (45, 46). Both Cer-16 and SM-16 have been associated with neurodegenerative diseases such as dementia including Alzheimer’s disease (22, 47–55), Huntington’s and Parkinson’s disease (56–59) as well as neuroinflammation (60, 61). Our study also showed that Cer-16 and SM-16, but not other species of Cer and SM, associated with higher levels of NfL in blood, which is a nonspecific biomarker for neuronal damage. Recent studies in CHS have shown that serum NfL and GFAP were associated with substantially higher risk of incident dementia and dementia mortality (62) and WMG worsening over 5 years (37). Altered sphingolipid metabolism may similarly reflects increased neurodegeneration, neuroinflammation or both and might be valuable as a biomarker in conjunction with NfL and GFAP. The reason for the differences observed in our findings regarding NfL and GFAP is unclear. However, our previous research has shown a tendency for NfL to exhibit a higher correlation with vascular brain injuries compared to GFAP in CHS (37).

Our findings provide crucial evidence that the direction of association of Cer and SM with several brain pathology outcome depends on the fatty acid attached to the sphingosine backbone. Our research demonstrates that elevated baseline plasma levels of Cer-16 and SM-16 are associated with a higher degree of brain pathology, while higher baseline plasma levels of Cer and SM with very-long chain fatty acids are independently inversely associated. Several cell and animal studies suggest that Cer have different biological properties dependent on the length of the fatty acid chain (25, 26). Diverse Cer can alter cell membrane properties, where fluidity increases with higher levels of Cer-16 and lower amounts of Cer-22 and Cer-24 (25, 63). The enzyme ceramide synthases 2 (CerS2) produces very-long chain ceramides and a study in CerS2 null mice showed that these mice had defective myelin sheath with lower levels of Cer-24 and higher levels Cer-16 (64). Another study of downregulation of CerS2 observed induced cell autophagy with a reduction in the levels of Cer-24 and increase in Cer-16 (65). Overexpression of ceramide synthases 6 (CerS6), which produces long-chain ceramides (C14-C18), increased the secretion of the inflammatory cytokine TNF-α in the liver (66). In addition, several studies report that Cer-16 promotes apoptosis, while Cer-22 and Cer-24 appear to protect from apoptosis (14, 25, 26, 67, 68). Most studies have investigated ceramides; however, ceramides and sphingomyelins are highly interrelated and can be generated from each other.

This study has several strengths, which involve the large sample size, and the comprehensive assessment of multiple complementary subclinical brain pathology measurements including two MRIs performed 5 years apart, enabling longitudinal analysis. We also included circulating neuronal biomarkers to complement the radiological findings. Several limitations include the study participants being older and primarily Caucasian, thus our findings may not generalize to other ethnicities or younger populations. Additionally, the MRI measurements in CHS were performed in the 1990s and did not include imaging of fractional anisotropy. Also, additional brain imaging studies, such as PET scanning for proteinopathies, were not performed.

Lastly, further studies are warranted to determine whether the timing of the measurements (midlife vs. late life) is important. The levels of sphingolipids change with age (69–71), thus multiple measurements over a long time will be needed to fully understand the dynamics of sphingolipid metabolism with relation to disease.

## Conclusion

In this study of older adults, Cer and SM differed in association with several measurements of subclinical brain pathology including number of brain infarcts, and NfL blood levels, which were dependent on the length of the fatty acid chain bound. Confirmation of these results in other studies with repetitive measurements of sphingolipids, MRI, levels of circulating NfL and GFAP is necessary to establish their role biomarkers of degenerative and vascular brain injury.

## Data availability statement

The data analyzed in this study is subject to the following licenses/restrictions: access to data can be done through CHS. Requests to access these datasets should be directed to CHS-NHLBI.org.

## Ethics statement

The studies involving humans were approved by the Institutional Review Board at University of Washington. The studies were conducted in accordance with the local legislation and institutional requirements. The participants provided their written informed consent to participate in this study.

## Author contributions

KFM: Conceptualization, Formal analysis, Investigation, Visualization, Writing – original draft, Writing – review & editing. JH: Writing – original draft, Writing – review & editing. AF: Writing – original draft, Writing – review & editing. LD: Writing – original draft, Writing – review & editing. WL: Writing – original draft, Writing – review & editing. OL: Writing – original draft, Writing – review & editing. AH: Writing – original draft, Writing – review & editing. MJ: Supervision, Writing – original draft, Writing – review & editing. RL: Writing – original draft, Writing – review & editing. KJM: Supervision, Writing – original draft, Writing – review & editing.
