# Supplementary material for: Circulating sphingolipids and subclinical brain pathology: the cardiovascular health study
Source: Front Neurol. 2024 May 3;15:1385623. doi: 10.3389/fneur.2024.1385623 (PMC11099203; doi:10.3389/fneur.2024.1385623)
Supplement: Supplementary file 1 [file Table_1.docx]

**Supplement**

**Supplement Table S1.** Characteristics of CHS participants with sphingolipid measurements in the different study populations based on outcome.

| **Characteristics** | Worsening in WMG | Worsening in VG | Number of brain infarcts | Quantitative measurements of brain volume | NfL and GFAP measurements |
| --- | --- | --- | --- | --- | --- |
| n | 1842 | 1801 | 1960 | 1008 | 2061 |
| Age, years | 75.7 (4.4) | 75.7 (4.4) | 75.6 (4.4) | 75.7 (4.3) | 75.8 (4.5) |
| Male | 752 (40.8%) | 738 (41.0%) | 785 (40.1%) | 424 (42.1%) | 817 (39.6%) |
| Black race | 285 (15.5%) | 282 (15.7%) | 292 (14.9%) | 127 (12.6%) | 284 (13.8%) |
| Education, years | 12.9 (3.0) | 12.9 (3.0) | 12.9 (2.9) | 13.2 (2.8) | 12.9 (3.0) |
| BMI, kg/m^2^ | 26.6 (4.2) | 26.6 (4.2) | 26.66 (4.2) | 26.69 (4.5) | 26.6 (4.3) |
| Physical activity, kcal/week | 1623 (1804) | 1627 (1805) | 1633 (1806) | 1698 (1870) | 1637 (1833) |
| Alcohol intake, drinks/week | 2.3 (5.3) | 2.2 (5.2) | 2.2 (5.2) | 2.6 (5.7) | 2.3 (5.5) |
| Current smoker | 151 (8.2%) | 147 (8.2%) | 158 (8.1%) | 82 (8.1%) | 165 (8.0%) |
| Depression score^*1^ | 4 [1, 7] | 4 [1, 7] | 4 [2, 7] | 4 [2, 7] | 4 [2, 7] |
| Diabetes | 208 (11.3%) | 210 (11.7%) | 221 (11.3%) | 113 (11.2%) | 56 (2.7%)^2^ |
| Coronary heart disease | 348 (18.9%) | 337 (18.7%) | 359 (18.3%) | 200 (19.8%) | 382 (18.5%) |
| Hypertension | 1019 (55.3%) | 996 (55.3%) | 1066 (54.4%) | 516 (51.2%) | 1089 (52.8%) |
| Lipid-lowering medication use | 134 (7.3%) | 132 (7.3%) | 143 (7.3%) | 73 (7.2%) | 149 (7.2%) |
| HDL cholesterol, mg/dL | 54.3 (14.6) | 54.2 (14.6) | 54.3 (14.6) | 53.8 (14.3) | 54.4 (14.4) |
| LDL cholesterol, mg/dL | 128 (32.1) | 127 (32.3) | 128 (32.4) | 128 (32.1) | 129 (32.8) |
| *APOE ε4* carrier | 422 (24.8%)^3^ | 414 (24.5%)^4^ | 447 (24.7%)^5^ | 236 (25.1%)^6^ | 488 (25.8%)^7^ |
| Sphingolipid concentration, μg/dL^*^ |  |  |  |  |  |
| Cer16 | 0.25 [0.22, 0.29] | 0.25 [0.22, 0.29] | 0.25 [0.22, 0.29] | 0.26 [0.22, 0.29] | 0.25 [0.22, 0.29] |
| Cer20 | 0.08 [0.06, 0.10] | 0.08 [0.06, 0.10] | 0.08 [0.06, 0.10] | 0.08 [0.06, 0.10] | 0.08 [0.06, 0.10] |
| Cer22 | 0.60 [0.49, 0.72] | 0.60 [0.49, 0.72] | 0.60 [0.49, 0.73] | 0.59 [0.48, 0.72] | 0.59 [0.49, 0.72] |
| Cer24 | 4.4 [3.8, 5.1] | 4.37 [3.77, 5.11] | 4.4 [3.8, 5.1] | 4.35 [3.76, 5.13] | 4.32 [3.73, 5.06] |
| SM16 | 123 [112, 134] | 123 [112, 134] | 123 [112, 134] | 121 [110, 133] | 123 [112, 134] |
| SM20 | 17.8 [15.6, 20.0] | 17.7 [15.6, 19.9] | 17.8 [15.6, 20.1] | 17.7 [15.5, 20.3] | 17.6 [15.4, 20.0] |
| SM22 | 26.6 [23.3, 30.4] | 26.5 [23.3, 30.4] | 26.5 [23.3, 30.4] | 26.1 [22.7, 29.7] | 26.5 [23.1, 30.3] |
| SM24 | 14.3 [12.2, 16.6] | 14.3 [12.2, 16.5] | 14.2 [12.1, 16.6] | 14.2 [12.1, 16.5] | 14.2 [12.1, 16.5] |
| No. with worsening in WMG change | 511 (27.7%) | 498 (27.8%) | 491 (27.6%) | 261 (25.9%) | 577 (28.0%) |
| No. with worsening in VG change | 523 (29.2%) | 525 (29.2%) | 499 (28.7%) | 277 (27.5%) | 585 (28.4%) |
| No. of MRI-defined infarcts |  |  |  |  |  |
| 0 | 1234 (67.0%) | 1212 (67.3%) | 1340 (68.4%) | 690 (68.5%) | 1422 (69.0%) |
| 1-2 | 491 (26.7%) | 476 (26.4%) | 512 (26.1%) | 261 (25.9%) | 521 (25.3%) |
| ≥3 | 117 (6.4%) | 113 (6.3%) | 108 (5.5%) | 56.4 (5.6%) | 117 (5.7%) |
| Quantitative MRI measurements, cm^3^ |  |  |  |  |  |
| Total brain volume | 1.0 (0.1) | 1.0 (1.0) | 1.0 (0.1) | 1.0 (1.0) | 1.0 (1.0) |
| Mean bilateral  hippocampal volume | 3.1 (0.9) | 3.1 (0.9) | 3.1 (0.9) | 3.1 (0.9) | 3.1 (0.9) |
| NfL and GFAP concentration, pg/mL^*^ |  |  |  |  |  |
| NfL | 25.6 [20.0, 33.8] | 25.6 [20.0, 33.8] | 25.6 [20.0, 33.8] | 25.6 [20.0, 33.8] | 25.8 [20.0, 34.8] |
| GFAP | 248 [187, 351] | 248 [187, 351] | 248 [187, 351] | 248 [187, 351] | 249 [190, 350] |

Values are presented as mean (SD) or n (%) unless noted otherwise. ^*^Values are presented as median [IQR]

Abbreviations: *APOE* *E4*, apolipoprotein E4; BMI, body mass index; Cer, ceramide; GFAP, glial fibrillary acidic protein; HDL, high-density lipoprotein; LDL, low-density lipoprotein; MRI, magnetic resonance imaging; NfL, neurofilament light chain; SM, sphingomyelins; VG, ventricular grade; WMG, white matter grade; 16, 20, 22, and 24 stand for the number of carbons of the saturated fatty acid acylated to the sphingolipid backbone.

^1^ Depression Score measured with the 10-item version of the Center for Epidemiological Studies Depression Scale with maximum of 30 possible points. ^2^ Participants had a fasting glucose concentration of ≥7 mmol/L (126 mg/dL) at sphingolipid measurements. ^3^ N = 1701. ^4^ N = 1688. ^5^ N = 1810. ^6^ N = 939. ^7^ N = 1892.

**Supplement Table S2.** Plasma concentrations and correlations of sphingolipids, N = 4612.

| Sphingolipid | CV, % | Mean (SD), μg/mL^1^ | Spearman correlation coefficient | | | | | | | |
| --- | --- | --- | --- | --- | --- | --- | --- | --- | --- | --- |
|  |  |  | Cer | | | | SM | | | |
|  |  |  | 16 | 20 | 22 | 24 | 16 | 20 | 22 | 24 |
| Cer-16 | 12.5 | 0.27 (0.06) | 1.00 |  |  |  |  |  |  |  |
| Cer-20 | 18.6 | 0.08 (0.03) | 0.51 | 1.00 |  |  |  |  |  |  |
| Cer-22 | 7.8 | 0.62 (0.18) | 0.60 | 0.61 | 1.00 |  |  |  |  |  |
| Cer-24 | 12.5 | 4.49 (1.08) | 0.59 | 0.50 | 0.87 | 1.00 |  |  |  |  |
| SM-16 | 5.9 | 125.0 (19.1) | 0.43 | 0.30 | 0.21 | 0.28 | 1.00 |  |  |  |
| SM-20 | 7.5 | 17.7 (3.56) | 0.30 | 0.37 | 0.46 | 0.48 | 0.49 | 1.00 |  |  |
| SM-22 | 6.7 | 26.6 (5.86) | 0.35 | 0.31 | 0.57 | 0.54 | 0.58 | 0.79 | 1.00 |  |
| SM-24 | 6.3 | 14.3 (3.51) | 0.29 | 0.11 | 0.40 | 0.49 | 0.54 | 0.66 | 0.89 | 1.00 |

Abbreviations Cer, ceramide; SM, sphingomyelins; 16, 20, 22, and 24 stand for the number of carbons of the saturated fatty acid acylated to the sphingolipid backbone.

**Supplement Table S3.** Adjusted odds ratios for increase in number of brain infarcts per SD higher plasma log-sphingolipid concentrations, N = 1960. Results of Models 1 and 2.

| **Sphingolipid** | **OR for increase in number of brain infarcts (95% CI)** | | | | | |
| --- | --- | --- | --- | --- | --- | --- |
|  | **Model 1** | **P-value** | | **Model 2** | **P-value** | |
|  |  | ***unadjusted*** | ***FDR*** |  | ***unadjusted*** | ***FDR*** |
| **Cer-16** | 1.09 (0.99, 1.20) | 0.09 | 0.18 | 1.06 (0.96, 1.18) | 0.24 | 0.39 |
| **Cer-20** | **1.18 (1.07, 1.31)** | **0.001** | **0.008** | **1.16 (1.04, 1.29)** | **0.004** | **0.03** |
| **Cer-22** | 1.10 (0.99, 1.22) | 0.07 | 0.18 | 1.08 (0.97, 1.21) | 0.16 | 0.32 |
| **Cer-24** | 1.03 (0.93, 1.13) | 0.60 | 0.80 | 0.99 (0.89, 1.10) | 0.86 | 0.86 |
|  |  |  |  |  |  |  |
| **SM-16** | 1.09 (0.99, 1.21) | 0.09 | 0.18 | 1.06 (0.94, 1.19) | 0.14 | 0.32 |
| **SM-20** | 1.00 (0.90, 1.12) | 0.94 | 0.94 | 0.96 (0.85, 1.09) | 0.48 | 0.64 |
| **SM-22** | 1.02 (0.92, 1.13) | 0.70 | 0.80 | 0.98 (0.87, 1.10) | 0.65 | 0.75 |
| **SM-24** | 0.96 (0.86, 1.07) | 0.44 | 0.70 | 0.91 (0.81, 1.02) | 0.07 | 0.29 |

Abbreviations Cer, ceramide; CI, confidence interval; FDR, false discovery rate; OR, odds ratio; SM, sphingomyelins; 16, 20, 22, and 24 stand for the number of carbons of the saturated fatty acid acylated to the sphingolipid backbone. Model 1 adjusted for age, sex, race/ethnicity, education, field center, year of blood sample measurement. Model 2 adjusted as for Model 1 in addition to BMI, physical activity, alcohol intake, smoking status, depression score, prevalent diabetes, prevalent coronary heart disease, prevalent hypertension, lipid-lowering medication use, HDL-cholesterol, and LDL-cholesterol.

**Supplement Table S4.** Cross-sectional difference in NfL serum levels per SD higher plasma log-sphingolipid concentrations, N = 2061.

| **Sphingolipid** | **β for NfL serum levels (95% CI)** | | | | | |
| --- | --- | --- | --- | --- | --- | --- |
|  | **Model 1** | **P-value** | | **Model 2** | **P-value** | |
|  |  | ***unadjusted*** | ***FDR*** |  | ***unadjusted*** | ***FDR*** |
| **Cer-16** | **0.03 (0.01, 0.06)** | **0.001** | **0.004** | **0.04 (0.01, 0.06)** | **0.001** | **0.009** |
| **Cer-20** | -0.01 (-0.03, 0.01) | 0.49 | 0.65 | -0.02 (-0.04, 0.004) | 0.12 | 0.30 |
| **Cer-22** | 0.002 (-0.02, 0.02) | 0.87 | 0.87 | 0.003 (-0.02, 0.03) | 0.77 | 0.83 |
| **Cer-24** | 0.01 (-0.01, 0.04) | 0.16 | 0.41 | 0.01 (-0.01, 0.04) | 0.23 | 0.30 |
|  |  |  |  |  |  |  |
| **SM-16** | **0.04 (0.02, 0.06)** | **<0.001** | **0.001** | 0.02 (-0.0002, 0.05) | 0.05 | 0.21 |
| **SM-20** | -0.01 (-0.04, 0.01) | 0.20 | 0.41 | -0.02 (-0.04, 0.01) | 0.19 | 0.30 |
| **SM-22** | -0.005 (-0.03, 0.02) | 0.68 | 0.78 | -0.02 (-0.04, 0.01) | 0.20 | 0.30 |
| **SM-24** | 0.01 (-0.01, 0.03) | 0.46 | 0.65 | -0.003 (-0.03, 0.02) | 0.83 | 0.83 |

Abbreviations Cer, ceramide; CI, confidence interval; FDR, false discovery rate; NfL, neurofilament light chain; SM, sphingomyelins; 16, 20, 22, and 24 stand for the number of carbons of the saturated fatty acid acylated to the sphingolipid backbone. Model 1 adjusted for age, sex, race/ethnicity, education, field center, year of blood sample measurement. Model 2 adjusted for Model 1 in addition to BMI, physical activity, alcohol intake, smoking status, depression score, prevalent diabetes, prevalent coronary heart disease, prevalent hypertension, lipid-lowering medication use, HDL-cholesterol, and LDL-cholesterol.

**Supplement Table S5.** Cross-sectional difference in NfL serum levels per SD higher plasma log-sphingolipid concentrations in *APOE* genotype stratified analyses. Total N = 1892.

| **Sphingolipid** | **β for NfL serum levels (95% CI)** | | | | | |
| --- | --- | --- | --- | --- | --- | --- |
|  | ***APOE ε4* non-carrier**  **(N = 1404)** | | | ***APOE ε4* carrier**  **(N = 488)** | | |
|  | **Model 2** | **P-value** | | **Model 2** | **P-value** | |
|  |  | ***unadjusted*** | ***FDR*** |  | ***unadjusted*** | ***FDR*** |
| **Cer-16** | 0.03 (0.003, 0.06) | 0.03 | 0.22 | 0.04 (-0.006, 0.08) | 0.08 | 0.39 |
| **Cer-20** | 0.002 (-0.03, 0.03) | 0.88 | 0.91 | -0.01 (-0.05, 0.03) | 0.51 | 0.94 |
| **Cer-22** | 0.01 (-0.02, 0.04) | 0.48 | 0.64 | 0.01 (-0.03, 0.05) | 0.65 | 0.94 |
| **Cer-24** | 0.02 (-0.01, 0.05) | 0.17 | 0.35 | 0.03 (-0.02, 0.07) | 0.21 | 0.56 |
|  |  |  |  |  |  |  |
| **SM-16** | 0.03 (-0.001, 0.06) | 0.05 | 0.22 | **0.07 (0.02, 0.11)** | **0.004** | **0.03** |
| **SM-20** | -0.02 (-0.045 0.01) | 0.17 | 0.34 | 0.01 (-0.04, 0.05) | 0.82 | 0.94 |
| **SM-22** | -0.01 (-0.04, 0.02) | 0.48 | 0.64 | 0.001 (-0.05, 0.05) | 0.97 | 0.97 |
| **SM-24** | 0.002 (-0.03, 0.03) | 0.91 | 0.91 | 0.006 (-0.04, 0.05) | 0.80 | 0.94 |

Abbreviations: APOE, apolipoprotein E; Cer, ceramide; CI, confidence interval; FDR, false discovery rate; OR, odds ratio; SM, sphingomyelins; 16, 20, 22, and 24 stand for the number of carbons of the saturated fatty acid acylated to the sphingolipid backbone. Model 2 adjusted for age, sex, race/ethnicity, education, field center, year of blood sample measurement, BMI, physical activity, alcohol intake, smoking status, depression score, prevalent diabetes, prevalent coronary heart disease, prevalent hypertension, lipid-lowering medication use, HDL-cholesterol, and LDL-cholesterol. *APOE ε4* non-carrier: ε2/ε2, ε2/ε3, or ε3/ε3; *APOE ε4* carrier: ε2/ε4, ε3/ε4, or ε4/ε4. SM-16: *P* for APOE genotype interaction *P* <0.001.
